# Author Correction: Data suggest COVID-19 affected numbers greatly exceeded detected numbers, in four European countries, as per a delayed SEIQR model

**DOI:** 10.1038/s41598-021-90076-y

**Published:** 2021-05-25

**Authors:** Sankalp Tiwari, C. P. Vyasarayani, Anindya Chatterjee

**Affiliations:** 1 Mechanical Engineering, Indian Institute of Technology Kanpur, Kanpur, 208016 India; 2 Mechanical and Aerospace Engineering, Indian Institute of Technology Hyderabad, Sangareddy, 502285 India

Correction to: *Scientific Reports* 10.1038/s41598-021-87630-z, published online 14 April 2021

This Article contains an error in Figure 3, where the top left panel is a duplication of the top right panel. The correct Figure 3 appears below as Figure 1.

**Figure 1:**
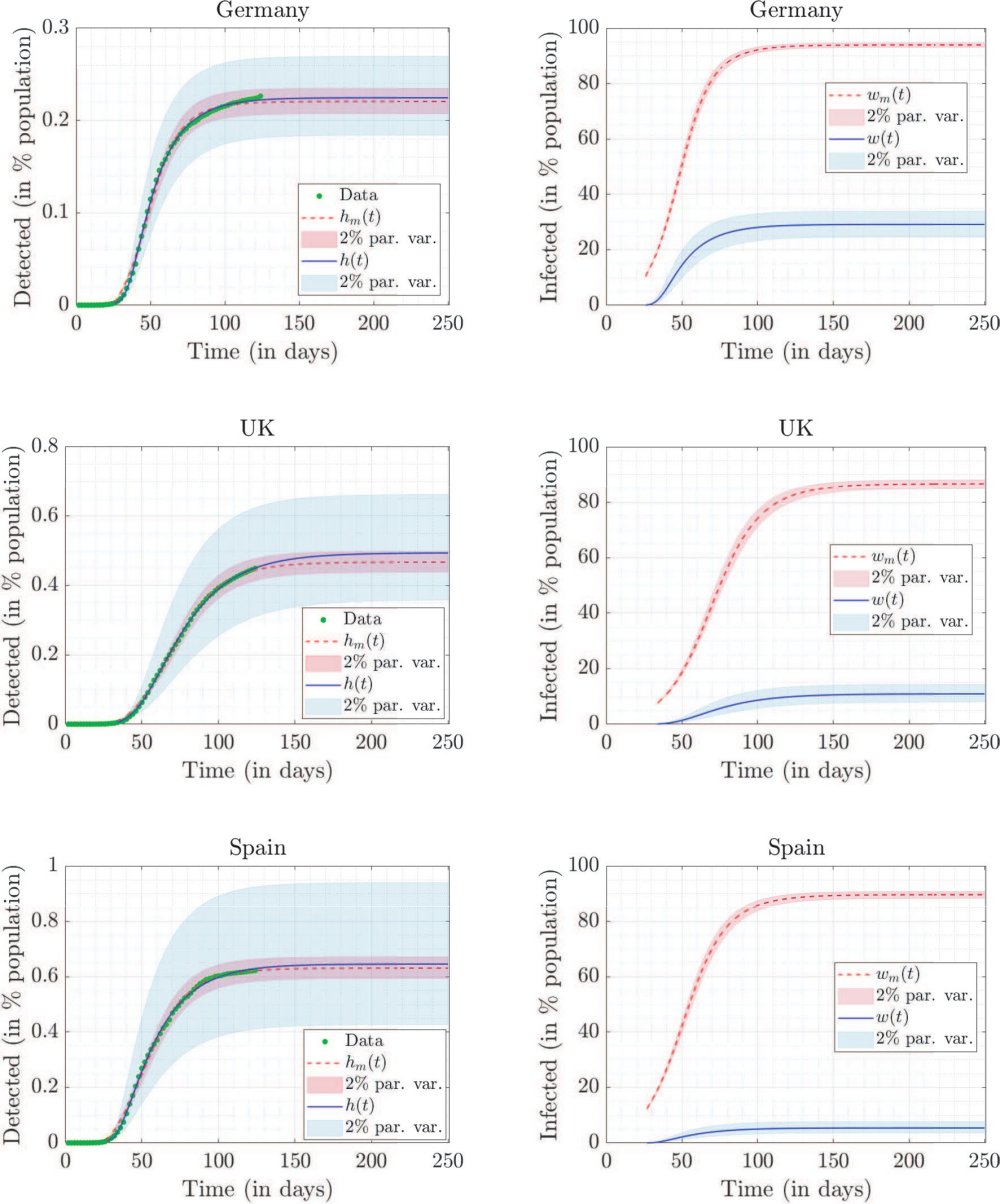
A correct version of the original Figure 3.

